# Supplementary material for: NLRC5 Deficiency Reduces LPS-Induced Microglial Activation via Inhibition of NF-κB Signaling and Ameliorates Mice’s Depressive-like Behavior
Source: Int J Mol Sci. 2023 Aug 26;24(17):13265. doi: 10.3390/ijms241713265 (PMC10487775; doi:10.3390/ijms241713265)
Supplement: Supplementary file 1 [file ijms-24-13265-s001.zip › Supplementary Figure.pdf]

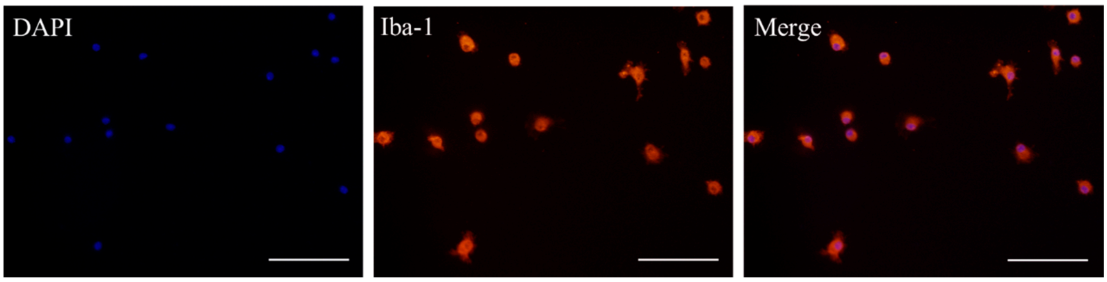

Supplementary Figure S1. Identification of primary cultured microglia.

Representative immunofluorescence images of primary cultured microglia (Blue: DAPI, Red: Iba-1, Scale bar: 100 μm).

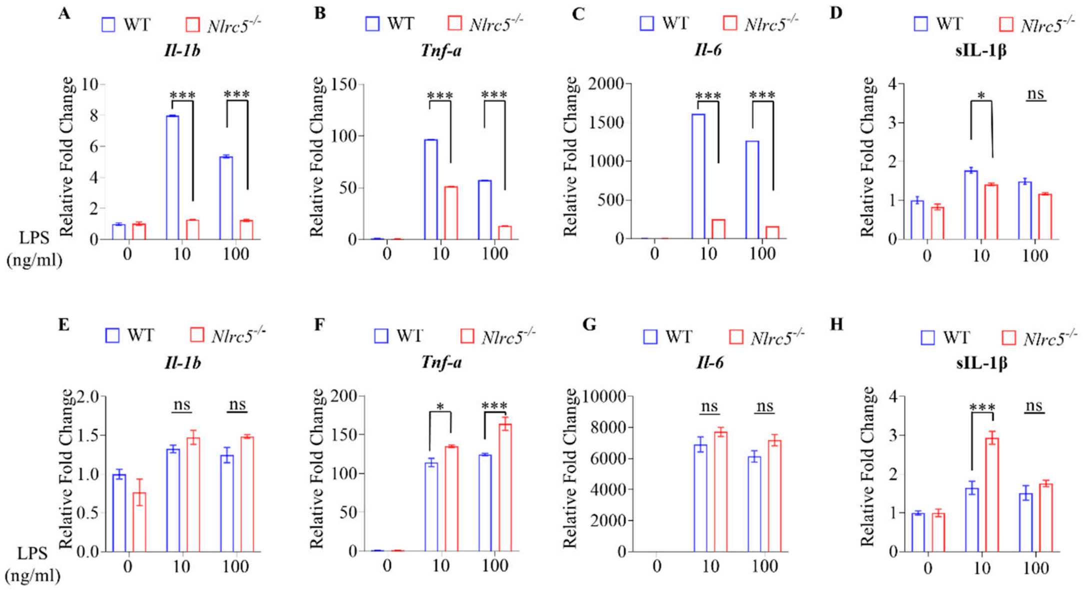

Supplementary Figure S2. *Nlrc5* knockout differently regulates cytokine production in primary cultured microglia and BMDM after treated with LPS for 8h.

(A-D) Effect of *Nlrc5* knockout on cytokine production in primary cultured microglia. The mRNA of *Il-1b* **(A)**, *Tnf-a* **(B)** and *Il-6* **(C)** of microglia treated with different concentration of LPS for 8 hours was detected by RT-qPCR and secreted IL-1β **(D)** in the supernatant was detected by ELISA (n=3 for each group). **(E-H)** Effect of *Nlrc5* knockout on cytokine production in BMDM. The mRNA of *Il-1b* **(E)**, *Tnf-a* **(F)** and *Il-6* **(G)** in BMDM treated with different concentration of LPS for 8 hours was detected by RT-qPCR and IL-1β secretion **(H)** in the supernatant was detected by ELISA (n=3 for each group). All data were presented as mean ± SEM. Two-way ANOVA was used to

determine the statistical significance between two group. *$p \leq 0.05$, **$p \leq 0.01$ and ***$p \leq 0.001$; ns: no significant difference. BMDM: bone marrow derived macrophages.

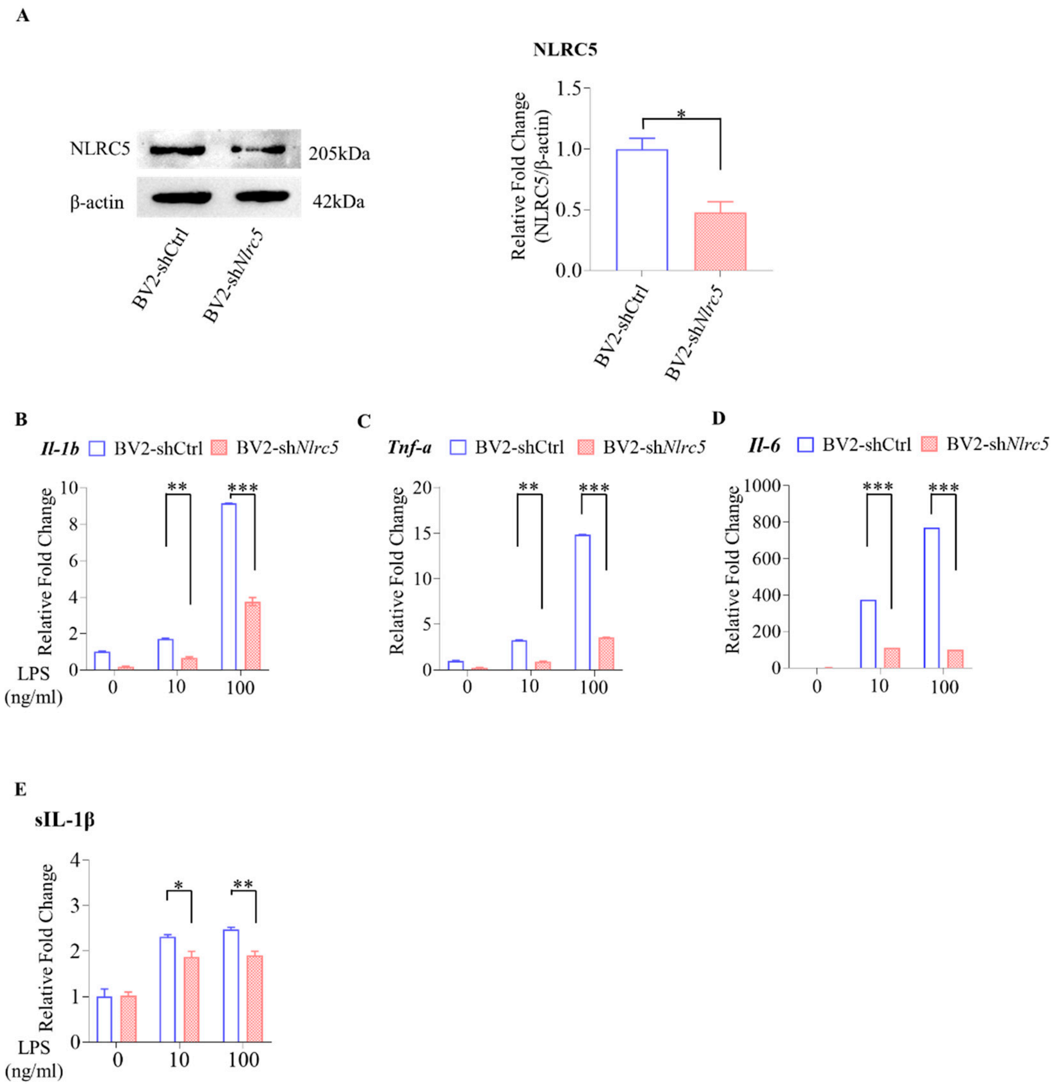

Supplementary Figure S3. Knockdown of *Nlrc5* reduced LPS-induced cytokines production in BV2.

(A) Confirmation the knockdown of *Nlrc5* by Western blot in BV2 cells. T test was used to determine the statistical significance between two groups. *$p \leq 0.05$. The mRNA of *Il-1b* **(B)**, *Tnf-a* **(C)** and *Il-6* **(D)** of BV2-shCtrl and BV2-sh*Nlrc5* treated with different concentration of LPS for 24 hours was detected by RT-qPCR and secreted IL-1β **(E)** in the supernatant was detected by ELISA (n=3 for each group). All data were presented as mean ± SEM. Two-way ANOVA was used to determine the statistical significance between two group. *$p \leq 0.05$, **$p \leq 0.01$ and ***$p \leq 0.001$.

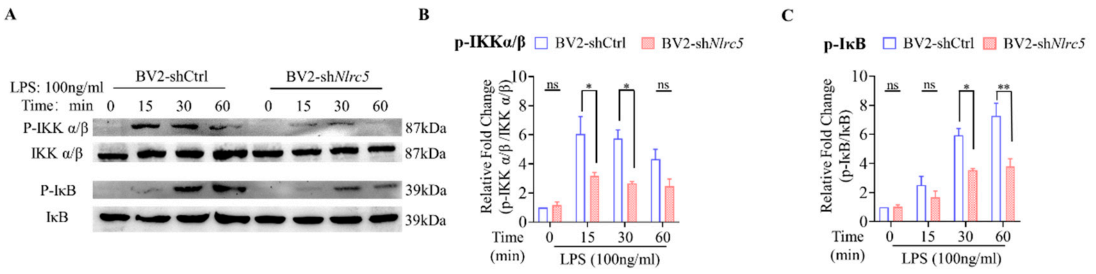

Supplementary Figure S4. Knockdown of *Nlrc5* inhibits IKK and IκB phosphorylation in BV2 cells.

(A) Representative Western blot image of p-IKKα/β, IKKα/β, p-IκB and IκB in BV2-shCtrl and BV2-sh*Nlrc5* cells treated with LPS (100 ng/mL) for different time. **(B,C)** The ratio of relative expression of p-IKKα/β to IKKα/β **(B)** and p-IκB to IκB **(C)** in LPS treated to untreated groups was quantified (n=4 for each group). All data were presented as mean ± SEM. Two-way ANOVA was used to determine the statistical significance between two group. *$p \leq 0.05$, **$p \leq 0.01$ and ***$p \leq 0.001$. ns: no significant difference.

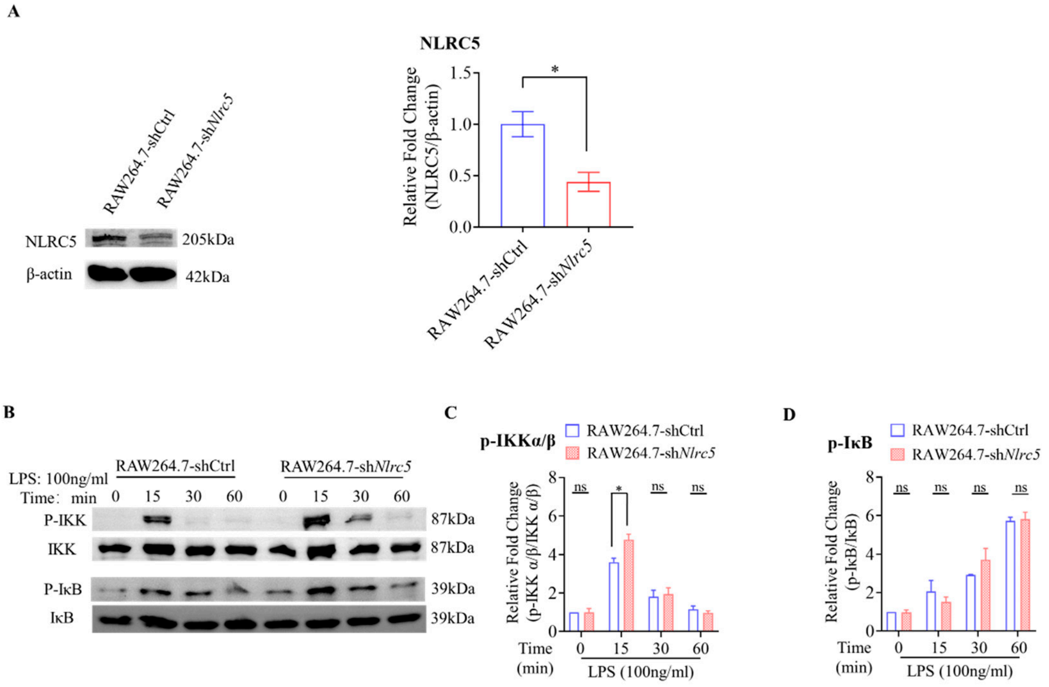

Supplementary Figure S5. Knockdown of *Nlrc5* promotes IKK and IκB phosphorylation in RAW264.7 macrophages.

(A) Confirmation the knockdown of *Nlrc5* by Western blot in RAW264.7 macrophages. **(B)** Representative Western blot image of p-IKKα/β, IKKα/β, p-IκB and IκB in RAW264.7-shCtrl and RAW264.7-sh*Nlrc5* cells treated with LPS (100 ng/mL) for

different time. **(C, D)** The ratio of relative expression of p-IKKα/β to IKKα/β **(C)** and p-IκB to IκB **(D)** in LPS treated to untreated groups was quantified (n=4 for each group). All data were presented as mean ± SEM. Two-way ANOVA was used to determine the statistical significance between two group. *p≤0.05, **p≤0.01 and ***p≤0.001. ns: no significant difference.

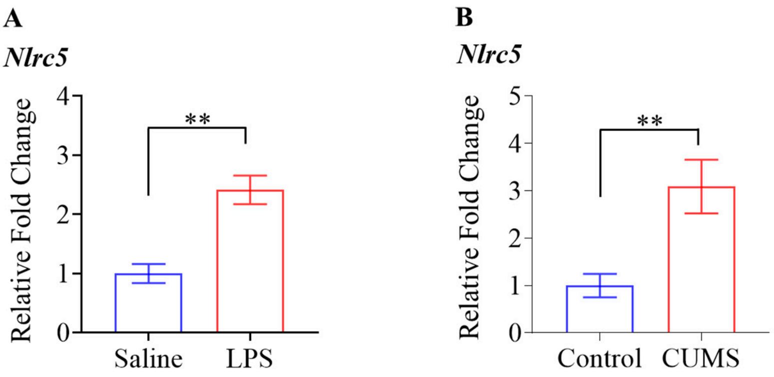

Supplementary Figure S6. *Nlrc5* was up-regulated in hippocampus tissue of mouse model of depression.

Expression of *Nlrc5* was assessed in hippocampus tissues of LPS **(A)** and CUMS **(B)** mice model by RT-qPCR (n=5 for each group). All data were presented as mean ± SEM. T test was used to determine the statistical significance between two groups. **p≤0.01. ns: no significant difference.

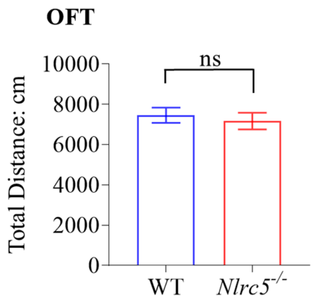

Supplementary Figure S7. Assessment of spontaneous locomotor ability in OPT.

Total travel distance of WT and *Nlrc5*$^{-/-}$ mice in open field test (n=12 for each group). All data were presented as mean ± SEM. T test was used to determine the statistical significance between two groups. ns: no significant difference.
