# Supplementary material for: NLRC5 Deficiency Reduces LPS-Induced Microglial Activation via Inhibition of NF-κB Signaling and Ameliorates Mice’s Depressive-like Behavior
Source: Int J Mol Sci. 2023 Aug 26;24(17):13265. doi: 10.3390/ijms241713265 (PMC10487775; doi:10.3390/ijms241713265)
Supplement: Supplementary file 1 [file ijms-24-13265-s001.zip › Supplementary Table 1.pdf]

Supplementary Table S1. The information of PCR primers.

List of oligonucleotide sequences	5’-3’
Tnf-a-F	AGTCCGGGCAGGTCTACTTT
Tnf-a-R	ACCCTGAGCCATAATCCCCT
Il-1b-F	ATGCCACCTTTTGACAGTGATG
Il-1b-R	GCAGCCCTTCATCTTTTGGG
Il-6-F	TCTATACCACTTCACAAGTCGGA
Il-6-R	GAATTGCCATTGCACAACTCTTT
β-actin-F	GTGACGTTGACATCCGTAAAGA
β-actin-R	GCCGGACTCATCGTACTCC
Nlrc5-F	TCAGCCCAGAACAAGTATCC
Nlrc5-R	TGGGCACAGACTTCCATTAG
